# Absence of blood parasites and other vector-borne pathogens in Alpine marmots (*Marmota marmota*) in Western Austria

**DOI:** 10.1007/s00436-020-07006-6

**Published:** 2021-01-16

**Authors:** Hans-Peter Fuehrer, Ilona Soukup, Bita Shahi-Barogh, Walter Glawischnig

**Affiliations:** 1 Institute of Parasitology, Department of Pathobiology, University of Veterinary Medicine Vienna, Veterinärplatz 1, 1210 Vienna, Austria; 2 Institute for Veterinary Disease Control Innsbruck, Austrian Agency for Health and Food Safety Ltd. (AGES), Technikerstrasse 70, 6020 Innsbruck, Austria

**Keywords:** Alps, Rodents, *Babesia*, *Anaplasma phagocytophilum*, Austria

## Abstract

The importance of vectors and vector-borne diseases (VBDs) is increasing on a global scale. Many vectors and pathogens benefit from global warming and can spread to novel habitats where they were formerly not present, including higher altitudes. Various vector-borne pathogens (VBPs), such as *Anaplasma phagocytophilum*, have been reported in, for instance, red foxes and wild ungulates in the Western Austrian Alps. However, these animals are known to migrate to lower regions in the winter season, and therefore, it is of interest to investigate if VBPs are also present in mammals faithful to their higher altitude alpine habitat all year round. Blood parasites and other VBPs, namely. Trypanosomatidae, piroplasms, *Hepatozoon* spp., filarioid helminths, Anaplasmataceae, and *Rickettisa* spp., were thus analysed with PCR in 148 alpine marmots (*Marmota marmota*). None of the marmots’ blood samples was positive for these VBPs, indicating a low abundance or absence of competent vectors in the alpine region. Alpine marmots seem to be naïve for VBPs (at least in our study area). An overview of VBD agents in other marmot species is given.

## Introduction

The impact of vector-borne diseases (VBDs) is increasing globally. VBDs are caused by various viruses, bacteria, protozoa, and helminths transmitted by competent vectors such as ticks, mosquitoes, sand flies, lice, and fleas. Lower temperatures at higher altitudes limit the development of VBDs and vector abundance (Zamora-Vilchis et al. 2012). Natural or human-driven environmental changes affect the ecological balance of hosts, vectors, and vector-borne pathogens (VBPs; Patz et al. 2000). Global warming influences the spread of VBPs and their vectors to areas where they were formerly not present. The increase of the daily average temperature (even in alpine areas) allows faster development of pathogens in vectors (e.g. filarioid helminths). Moreover, moderate winter temperatures lead to the spread of vectors not only to northern regions (in the Northern hemisphere) but also to areas with higher altitudes. Wildlife plays an important role in establishment of vectors and VBDs (Tomassone et al. 2018). Recently, several studies have documented the presence of VBPs (such as *Anaplasma phagocytophilum* and various *Babesia* spp.) in wildlife from Alpine regions in Western Austria, for instance, red foxes, red deer, chamois, and Alpine ibex (Cézanne et al. 2017; Hodžić et al. 2018; Messner et al. 2019). However, wildlife analysed in previous studies is known to migrate from higher to lower altitudes in the winter season, and so might be exposed to vectors while at the lower altitudes. Information on the occurrence of VBDs in a mammal faithful to its alpine habitat is therefore of high interest.

The Alpine marmot (*Marmota marmota*) is a sciurid rodent distributed in the Alps (including the western parts of the Austrian Alps), Tatras, Carpathians, and Apennines and was reintroduced into various regions such as the Eastern Alps and Pyrenees (Preleuthner et al. 1995; Arnold 1999). The Alpine marmot is a remnant of the Pleistocene cold steppe, perfectly adapted to a colder climate (shown by its habit of hibernating and its use of burrows). Alpine marmots have low levels of genetic variation (among the lowest of all mammals), and climate-adapted life history traps show low genetic diversity (Gossmann et al. 2019). It can be found in a range of alpine altitudes, from the tree line up to 3000 m altitude. They are normally not documented at altitudes below 900 m (Preleuthner et al. 1995). This social ground-dwelling squirrel species is mainly herbivorous, living in alpine meadows in large groups. Knowledge regarding the parasite fauna in Alpine marmots is limited to intestinal helminths and protozoa and certain ectoparasites (Preleuthner et al. 1999). Four marmot-specific intestinal helminths are common in *M. marmota*-*Ctenotaenia marmotae*, *Ascaris laevis*, *Citellina alpina*, and *Paranoplocephala transversaria* (Preleuthner et al. 1999; Callait and Gauthier 2000). Zanet et al. (2017) reported a higher risk of gastrointestinal helminth infections at lower elevations in Alpine marmots. Moreover, several coccid protozoa such as *Eimeria* spp., *Sarcocystis* spp., and *Toxoplasma gondii* were documented (Preleuthner et al. 1999; Richter et al. 2007). Only very few ectoparasites were reported from *M. marmota*, namely *Echinonyssus blanchardi*, *Neotrombicula autumnalis*, and rarely *Ixodes ricinus* ticks (Preleuthner et al. 1999). Non-parasitic oribatids act as intermediate hosts of *C. marmotae*, which might be transported into burrows with nesting material (Ebermann 1976; Arnold and Lichtenstein 1993). The most important ectoparasite of Alpine marmots is *E. blanchardi*. Ticks were rarely documented because they are normally not present in the Alpine marmots’ habitats (Alpine pastures above 1000 m). *Ix. ricinus* was found in *M. marmota* populations living in lower regions (Arnold and Lichtenstein 1993). However, in the past decade, *Ix. ricinus* was also regularly observed at altitudes up to and above 1700 m (personal communication, Gernot Walder; Messner et al. 2019; Garcia-Vozmediano et al. 2020).

At present, there is virtually no information regarding blood parasites and other VBPs in Alpine marmots. The aim of this study was to investigate the presence of various blood parasites and pathogens known to be present in wildlife in Western Austria, as well as VBDs described in other marmot species.

## Materials and methods

A total of 148 blood samples were collected during the hunting season of 2018 (August 5–September 30) from Alpine marmots (*Marmota marmota*) in the Western Austrian provinces Tyrol and Vorarlberg. Whenever possible, age, sex, and additional remarks (such as intestinal helminths) were registered by the hunters. From each marmot, a blood sample (approx. 50 μl) was spotted on Whatman-filter paper (VWR International GmBH, Vienna, Austria), air dried, and stored at room temperature. 4 × 4 mm pieces of filter paper were cut out of the centre of the blood spot and DNA extraction processed as reported previously using an InstaGene™ matrix kit (Bio-Rad Laboratories; Fuehrer et al. 2010). Samples were analysed for the presence of DNA of following vector-borne pathogens—*Babesia* and *Hepatozoon* (18S rRNA; BTH-1F/BTH-1R; GF2/GR2), Trypanosomatida (18S rRNA; S762F/S763R; TRnSSU-F2/TRnSSU-R2), filarioid nematodes (mt COI; COIint-F/COIint-R), Anaplasmataceae (16S rRNA; EHR16SD/EHR16SR), and *Rickettsia* spp. (23S/5S rRNA; ITS-F/ITS-R) according to published protocols (Maslov et al. 1996; Brown et al. 2001; Casiraghi et al. 2001; Vitorino et al. 2003; Zintl et al. 2011; Seward et al. 2017; Messner et al. 2019). PCR products were analysed on 2% agarose gels stained with Midori Green Advance (Nippon Genetics Europe, Germany). PCR products showing bands were purified and sequenced at LGC Genomics GmbH (Germany).

## Results and discussion

Blood samples of a total of 148 Alpine marmots were included in this study. Of those, 102 were collected in Tyrol and 46 in Vorarlberg (Table 1). Intestinal helminth infestations were noted in five animals. Moreover, one Alpine marmot presented with a systemic toxoplasmosis at histological examination.

**Table 1:** Sex, age class, and origin of Alpine marmots analysed in Western Austria

	District	Male	Female	Juvenile	Total
Tyrol	Imst	17	6	1	24
	Innsbruck-Land	18	14	2	34
	Landeck	15	5	0	20
	Lienz	15	5	3	23
	Schwaz	0	1	0	1
Vorarlberg	Bludenz	24	15	1	40
	Bregenz	1	3	1	6*
	Total	90	49	8	148

*The sex/age of one marmot was unknown

None of the 148 Alpine marmots was positive for Anaplasmataceae, *Rickettsia* spp., *Babesia* spp., *Hepatozoon* spp., Trypanosomatida, or filarioid helminths. This is of special interest, since this demonstrates the absence or low abundance of vectors in the study area at > 1000 m above sea level.

In contrast, other marmot species—especially those living at lower altitudes—are common hosts of vector-borne pathogens.

Fifteen species of the genus *Marmota* occur in Asia, Europe, and Northern America (Herron et al. 2004; Brandler and Lyapunova 2009). Some species live in mountainous areas; others prefer rough grassland (and are so more prone to vector contact). Several VBD studies have been conducted in two North American marmot species—the groundhog (*M. monax*) and yellow-bellied marmot (*M. flaviventris*).

The groundhog or woodchuck (*M. monax*) is a North American marmot species preferring open country and edges of woodland. The parasite fauna and other vector-borne pathogens of groundhogs are well studied, and these animals are also used as laboratory animals (Fleming et al. 1979; Hilken et al. 2003). Various intestinal parasites but also ectoparasites are documented (Crouch 1936). *Ix. cookei*, *Ix. hexagonus*, *Ix. dammini*, and *Dermacentor variabilis* were found on *M. monax* (Crouch 1936; Magnarelli et al. 1991; Smith et al. 2019). *Ixodes cookei* ticks (so called groundhog ticks) are believed to play a role in the transmission of Powassan virus lineage 1 (Mlera and Bloom 2018). Groundhogs are thought to be potential wildlife reservoirs of *Borrelia burgdorferi*, but *Ix. cookei* ticks have been shown to be ineffective vectors for this pathogen (Barker et al. 1993). A study conducted in Pennsylvania (USA) reported 58% of 659 woodchucks sero-positive for *Ehrlichia chaffeensis* (Nicholson et al. 2003). Moreover, woodchucks are exposed to spotted fever group rickettsiae (Magnarelli et al. 1991). Reeves et al. (2005) reported *Rickettsia typhi* in *Enderleinellus marmotae* (host-specific louse) collected from *M. monax* in SC, USA. The vector capacity of *E. marmotae* for *R. typhi* remains unclear because several flea species including *Pulex irritans* feed on groundhogs (Reeves et al. 2005).

*Ackertia marmotae* is an onchocercid nematode in the liver of woodchucks. It is transmitted by *Ix. cookei*. In Tompkins County, New York (USA), this parasite was found in 78% (151 of 194) of woodchucks (Fleming et al. 1979). *A. marmotae* is able to survive hibernation as 4th stage larvae (Fleming et al. 1979). Moreover, *Hepatozoon* sp. was documented in the whole blood of a single woodchuck in MO, USA (Allen et al. 2011).

The yellow-bellied marmot is native to mountainous areas in the USA and Canada and is found between 1600 and 4250 m above sea level (Armstrong et al. 2010). In a study conducted in Yosemite National Park in California, Fleer et al. (2011) serologically screened one yellow-bellied marmot for various VBDs, and it tested positive for *An. phagocytophilum* (negative for *Borrelia* and *Rickettsia* spp.). *Trypanosoma* sp. was reported in *M. flaviventer nosophora* (Dias 1938) and yellow-bellied marmots in Colorado (Nouri and Blumstein 2019). Lopez et al. (2013) documented flea- and louse-transmitted *T. lewisi.* They stated that age is significantly associated with infection, and yearlings are more likely to be infected.

Although marmots are associated with important pathogens such as *Yersina pestis*, there is a lack of knowledge about vector-borne pathogens in other marmot species. Various fleas of the genus *Citellophilus* (some species are vectors of *Y. pestis*) parasitize marmots and ground squirrels (Medvedev et al. 2019). *Ix. autumnalis* was found on the Eurasian bobac marmot (*M. bobac*; Crouch 1936). The argasid tick *Ornithodoros* (*Ornithodoros*) *huajianensis* sp. nov. was described to be a parasite of the Mongolian marmot (*M. bobak sibirica*) in Gansu province, China (Sun et al. 2019). *Walchia marmota* sp. nov. is a trombiculid mite which parasitizes the Himalayan marmot (*M. himalayana*; Wen and Zhou 1997).

Overall the summary of various vector-borne pathogens and potential vectors shows that the genus *Marmota* harbours various VBDs. *M. marmota* seems to be naïve for VBDs, which might have a negative impact on Alpine marmot populations if vectors spread to higher altitudes. In contrast to migrating wildlife such as red foxes and wild ungulates, the Alpine marmot will be able to act as an indicator species to ascertain if certain vectors and vector-borne pathogens (e.g. *An. phagocytophilum*) have spread to alpine altitudes in the future.
